# The G Protein-Coupled Estrogen Receptor (GPER) Expression Correlates with Pro-Metastatic Pathways in ER-Negative Breast Cancer: A Bioinformatics Analysis

**DOI:** 10.3390/cells9030622

**Published:** 2020-03-04

**Authors:** Marianna Talia, Ernestina Marianna De Francesco, Damiano Cosimo Rigiracciolo, Maria Grazia Muoio, Lucia Muglia, Antonino Belfiore, Marcello Maggiolini, Andrew H. Sims, Rosamaria Lappano

**Affiliations:** 1 Department of Pharmacy, Health and Nutritional Sciences, University of Calabria, 87036 Rende, Italy; marianna.talia@unical.it (M.T.); damianocosimo.rigiracciolo@unical.it (D.C.R.); mariagraziamuoio@libero.it (M.G.M.); lucia.muglia@unical.it (L.M.); rosamaria.lappano@unical.it (R.L.); 2 Endocrinology, Department of Clinical and Experimental Medicine, University of Catania, Garibaldi-Nesima Hospital, 95122 Catania, Italy; ernestinamarianna@yahoo.it (E.M.D.F.); antonino.belfiore@unict.it (A.B.); 3 MRC Institute of Genetics and Molecular Medicine, University of Edinburgh, Edinburgh EH4 2XR, UK

**Keywords:** bioinformatics, GPER, breast cancer, TCGA, METABRIC, cell adhesion molecules, extracellular matrix, focal adhesion

## Abstract

The G protein-coupled estrogen receptor (GPER, formerly known as GPR30) is a seven-transmembrane receptor that mediates estrogen signals in both normal and malignant cells. In particular, GPER has been involved in the activation of diverse signaling pathways toward transcriptional and biological responses that characterize the progression of breast cancer (BC). In this context, a correlation between GPER expression and worse clinical-pathological features of BC has been suggested, although controversial data have also been reported. In order to better assess the biological significance of GPER in the aggressive estrogen receptor (ER)-negative BC, we performed a bioinformatics analysis using the information provided by The Invasive Breast Cancer Cohort of The Cancer Genome Atlas (TCGA) project and Molecular Taxonomy of Breast Cancer International Consortium (METABRIC) datasets. Gene expression correlation and the statistical analysis were carried out with R studio base functions and the tidyverse package. Pathway enrichment analysis was evaluated with Kyoto Encyclopedia of Genes and Genomes (KEGG) pathway on the Database for Annotation, Visualization and Integrated Discovery (DAVID) website, whereas gene set enrichment analysis (GSEA) was performed with the R package phenoTest. The survival analysis was determined with the R package survivALL. Analyzing the expression data of more than 2500 primary BC, we ascertained that GPER levels are associated with pro-migratory and metastatic genes belonging to cell adhesion molecules (CAMs), extracellular matrix (ECM)-receptor interaction, and focal adhesion (FA) signaling pathways. Thereafter, evaluating the disease-free interval (DFI) in ER-negative BC patients, we found that the subjects expressing high GPER levels exhibited a shorter DFI in respect to those exhibiting low GPER levels. Overall, our results may pave the way to further dissect the network triggered by GPER in the breast malignancies lacking ER toward a better assessment of its prognostic significance and the action elicited in mediating the aggressive features of the aforementioned BC subtype.

## 1. Introduction

Breast cancer (BC) is the most frequently diagnosed tumor worldwide (24.2%) and the leading cause of cancer death among females (14.5%) [1]. The breast malignancies encompass diverse subtypes (Luminal A, Luminal B, Her2-enriched, Triple-negative/basal-like, and Normal-like) that are characterized by peculiar gene expression profiles, biological features, and clinical outcomes [2]. Estrogens play a pivotal role in numerous physiological conditions; however, the action of these steroids is also extensively associated with an increased risk of BC development [3]. The estrogen signaling is mainly mediated by the estrogen receptor (ER)α and ERβ that, upon ligand activation, regulate the expression of target genes involved in cell growth, invasion, and survival [4]. A growing body of data has also evidenced that the seven-transmembrane G protein-coupled estrogen receptor (GPER, previously known as GPR30) can mediate the estrogen action in diverse normal and malignant cell contexts, including BC [5]. The activation of GPER triggers diverse transduction pathways including the epidermal growth factor receptor (EGFR), phosphatidylinositol 3-kinase/protein kinase B (PI3K/Akt), and mitogen-activated protein kinases (MAPKs) toward transcriptional and biological responses driving the progression of BC [5,6,7,8]. In this regard, we recently found that GPER mediates the activation of the focal adhesion kinase (FAK) and the formation of focal adhesions (FAs) in triple negative BC cells (TNBC), thus contributing to the acquisition of aggressive features by breast malignancies [9]. In accordance with these data, immunohistochemical studies on breast tumors have shown that the expression of GPER is correlated with increased tumor size, distant metastasis, and recurrence [10,11]. Conversely, Martin and co-workers demonstrated that low expression levels of GPER are associated with aggressive features in a large cohort of primary invasive BC patients [12]. These controversial observations may deserve further investigations in order to better assess prognostic and therapeutic values of GPER, particularly in BC.

In the last years, high throughput techniques have been employed in handling and extracting meaningful information from large multiomic datasets [13]. In particular, big data analysis methods and classification techniques have allowed accurate and comprehensive examination of global gene expression profiles [14]. As cancer genomic datasets incessantly grow in terms of size and complexity, the availability of accessible computational resources may facilitate rapid and cost-effective analysis toward new discoveries in cancer biology and profiling [15]. The Cancer Genome Atlas (TCGA) project, initiated in 2005 by the National Cancer Institute, aims to collect genomic alterations implicated in cancer using genome analysis technologies [16]. Furthermore, the Molecular Taxonomy of Breast Cancer International Consortium (METABRIC) is a Canada-United Kingdom project integrating genomic and transcriptomic profiles of a large cohort of primary breast tumors (over two thousand) along with long-term clinical follow-up [17]. In particular, systematic advances in cancer genomics provided by both the TCGA and METABRIC databases have contributed to highlight similarities and differences in the genomic architecture of the breast malignancy as well as to identify new candidate biomarkers and drug tumor targets toward tailored therapeutic strategies [17,18,19]. Here, we focused on data extracted from both TCGA and METABRIC datasets in order to better understand the role exerted by GPER as well as its prognostic value in the aggressive BC subtype lacking ER. Indeed, our analysis provides new insights regarding the association of pro-metastatic pathways with GPER in the ER-negative BC, therefore opening a new scenario for subsequent studies aimed to better evaluate its role in breast tumors characterized by a worse prognosis.

## 2. Materials and Methods

### 2.1. Data Source

Data from the publically available TCGA and METABRIC datasets were used in the current study. mRNA expression data (RNA Seq V2 RSEM) and associated clinical information reported in the Invasive Breast Cancer Cohort of the TCGA project were retrieved on 4 November 2019 from cBioPortal for cancer genomics (http://www.cbioportal.org/) as well as microarray gene expression data (Log2 transformed intensity values) and clinical information of the METABRIC cohort. Patients of both TCGA (N = 817) and METABRIC (N = 1980) were classified on the basis of the presence or the absence of ER (detected by immunohistochemistry). Gene expression and clinical information were filtered for missing values. The filtering resulted in 775 patients of TCGA and 1904 of METABRIC, which were used for the subsequent analysis.

### 2.2. Correlation Analysis

The Pearson correlation coefficients (*r*-values) between the expression levels of GPER and the other genes of the TCGA (N = 36877) and METABRIC (N = 24367) datasets were assessed in ER-negative BC patients using the *cor.test()* function and setting the method as “Pearson” in R Studio (version 3.6.1). The first 1000 most correlated genes of each dataset were intersected with the *intersect()* function in order to obtain the most correlated genes shared by the two datasets. The statistical analysis was performed by using the t-tests, and *p* < 0.001 was considered statistically significant.

### 2.3. Pathway Enrichment Analysis

In order to obtain the enrichment results of the selected genes into significant pathways, we uploaded our lists on the Database for Annotation, Visualization and Integrated Discovery (DAVID) functional annotation analysis website [20]. We analyzed a list of the 277 top GPER-correlated genes of the ER-negative patients shared by TCGA and METABRIC, choosing the official gene symbol as “select identifier” and gene list as “list type” in the options of the upload and selecting a limit species of “*Homo sapiens*” in the background. Selecting the functional annotation tool and the option of pathways, crucial pathways were obtained by KEGG pathway of DAVID and their numbers in the KEGG database.

### 2.4. Gene Set Enrichment Analysis (GSEA) 

GSEA was performed using the *gsea()* function of the phenoTest package (https://bioconductor.org/packages/release/bioc/html/phenoTest.html) in R Studio in order to test the association between the predefined groups of genes and a specific phenotype. The gene lists used for this analysis, derived from DAVID functional annotation tool, are the CAMs pathway (KEGG entry = hsa04514), the ECM-receptor interaction pathway (KEGG entry = hsa04512), and the FA signaling pathway (KEGG entry = hsa04510). We ranked the genes in accordance with the differential expression within GPER high and low (median expression value as threshold assessment) samples in the ER-negative subgroup of BC patients, verifying if the selected set of genes were enriched at the bottom or the top of the ranked list. We calculated the enrichment score (ES) that reflects the degree to which a set of genes is overrepresented at the extremes (top or bottom) of the entire ranked list. The magnitude of the increment depends on the correlation of one gene with the phenotype. In this analysis, 20,000 simulations were used (B = 20,000). *p* < 0.05 was considered significant.

### 2.5. Survival Analysis

Comprehensive survival analysis was conducted using TCGA gene expression data of GPER along with the DFI information; patients were filtered for missing values, and the ER and the HER2 statuses were used to divide the population. The survivALL package was employed to examine Cox proportional hazards for all possible points-of-separation (low-high cut-points), selecting the cut-point with the lowest *p*-value [21] and separating the patients into high (N = 27) and low (N = 93) GPER expression levels. The Kaplan–Meier survival curves were generated using the survival and the survminer packages.

### 2.6. Statistical Analysis

In this study, the analyses, including the t-test, and the scatter plots were performed with the R tydiverse package. *p*-values < 0.05 were considered significant. Heatmaps were performed with the R pheatmap package. Gene expression values of both TCGA and METABRIC datasets were normalized by calculating their respective normalized z-scores.

## 3. Results

**Figure 1:**
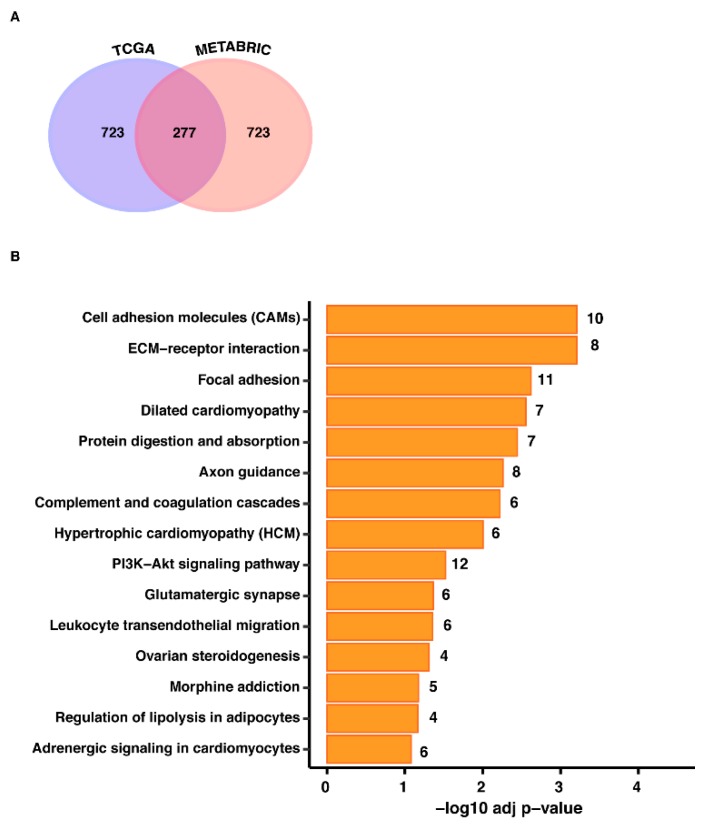
(**A**) Intersection of the top 1000 G protein-coupled estrogen receptor (GPER) correlated genes in estrogen receptor (ER)-negative breast cancer (BC) patients querying The Cancer Genome Atlas (TCGA) and Molecular Taxonomy of Breast Cancer International Consortium (METABRIC) datasets. (**B**) GPER expression is correlated with pro-metastatic pathways in ER-negative BC samples, as evaluated by The Kyoto Encyclopedia of Genes and Genomes (KEGG) pathway enrichment analysis of the 277 genes shared by the TCGA and METABRIC datasets and their positive correlation with GPER in ER-negative BC patients. The −log10 adjusted values are displayed along the x-axis, while the different KEGG pathways are shown along the y-axis. The number of the genes included in the identified pathways is plotted on the right of each bar.

Considering that GPER-mediated signaling has been involved in BC development and aggressiveness [6,7,19], we began our study correlating the expression of GPER with the genes present in both TCGA and METABRIC datasets. In particular, we focused our investigations on ER-negative BC, as this malignancy is characterized by a worse prognosis [1,2,19]. To this end, we ranked the genes by Pearson correlation coefficient, assessing for the next evaluations the first 1000 genes positively correlated with GPER either in TCGA or METABRIC cohorts. Hence, we found 277 shared genes between the two datasets, as shown in Figure 1A and detailed in the Supplementary Table S1.

In order to investigate the biological significance of the aforementioned 277 genes, we then performed KEGG (The Kyoto Encyclopedia of Genes and Genomes) pathway analysis using the online Database for Annotation, Visualization and Integrated Discovery (DAVID, http://david.abcc.ncifcrf.gov). The 277 genes were enriched in a number of pathways, as schematically shown in Figure 1B. Of note, transduction pathways that characterize aggressive cancer features as cell adhesion molecules (CAMs), extracellular matrix (ECM)-receptor interaction, and focal adhesion (FA) appeared to be the most significant, as indicated by their respective -log10 adj p-value.

**Figure 2:**
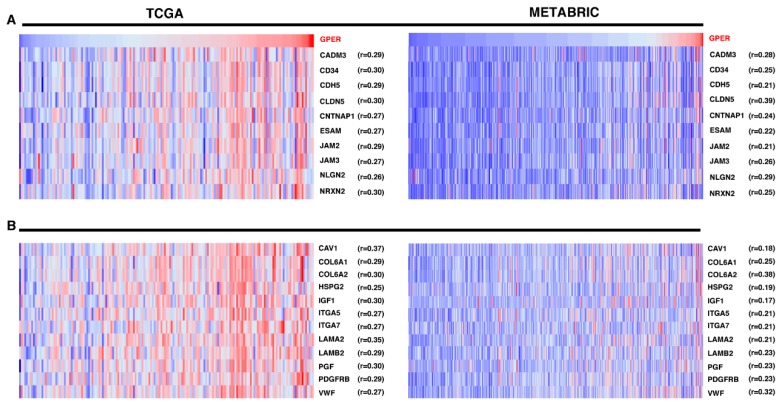
GPER correlates with the expression of cell adhesion molecules (CAMs), extracellular matrix (ECM)-receptor interaction, and focal adhesion (FA) pathway genes as determined querying the TCGA and the METABRIC datasets. The heatmaps, ranked from left to right, show the most GPER correlated genes belonging to the CAMs pathway (**A**) and to the ECM-receptor interaction and FA molecular pathways (**B**) in ER-negative breast tumor samples. Colors are z-score normalized values, red indicates high and blue indicates low.

Thereafter, we performed gene set enrichment analysis (GSEA) to explore the expression profile of the genes belonging to the CAMs, ECM-receptor interaction, and the FA pathways in the high and low GPER phenotypes of TCGA and METABRIC cohorts of ER-negative BC patients. It is worth noting that the genes included in these signaling pathways were found enriched in the group of patients showing high GPER levels (Supplementary Figure S1). In addition, we assessed the profile of the most GPER-correlated genes shared by the TCGA and METABRIC datasets. In this regard, we identified pro-tumorigenic genes belonging to the CAMs pathway as for instance the cell adhesion molecule 3 (*CADM3*), the CD34 molecule (*CD34*), the cadherin 5 (*CDH5*), the claudin 5 (*CLDN5*), the endothelial cell adhesion molecule (*ESAM*) and the junctional adhesion molecules namely *JAM2* and *JAM3* (Figure 2A). In addition, we evidenced further pro-tumorigenic genes belonging to the ECM-receptor interaction and FA pathways including for instance the caveolin 1 (*CAV1*), the alpha(α)1(VI) and the alpha(α)2(VI) chain of type VI collagen (*COL6A1* and *COL6A2,* respectively), the insulin like growth factor 1 (*IGF1*), the integrin subunit alpha 5 and the integrin subunit alpha 7 (*ITGA5* and *ITGA7,* respectively), the laminin subunit beta 2 (*LAMB2*), the platelet derived growth factor receptor beta (*PDGFRB*), the placental growth factor (*PGF*) and the von Willebrand factor (*VWF*) (Figure 2B).

**Figure 3:**
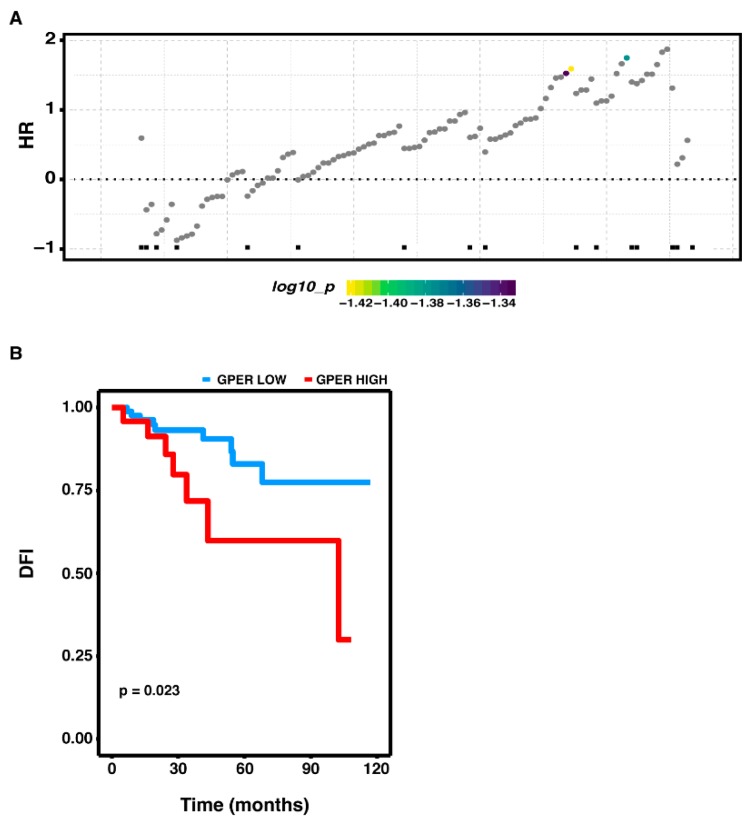
Clinical outcome on the basis of GPER expression in ER-negative BC patients. (**A**) ER-negative BC patients of the TCGA cohort divided into high and low expression levels of GPER on the basis of the established cut-point. The color bar gradient stands for range of the most significant points-of-separation of the population (low-high significance = blue-yellow gradient) based on GPER expression and survival of each patient. The x-axis represents the patients ordered by the increasing expression of GPER. (**B**) Correlation between GPER expression and disease free interval (DFI) of ER-negative BC patients in the TCGA cohort.

Next, we evaluated whether the expression of GPER would be predictive for the outcome of the aggressive BC subtype characterized by the lack of both ER the human epidermal growth factor receptor 2 (HER2). Using the disease free interval (DFI) data, a significant cut-point was predictable only from the TCGA cohort. Ranking the gene expression data according to the low and high GPER levels, all possible points-of-separation and their significance were reported in the survivALL plot by which the most significant cut-point was assessed (Figure 3A). Thereafter, the Kaplan-Meier survival curve revealed that a worse DFI characterizes the group of BC patients exhibiting a high expression of GPER (Figure 3B).

## 4. Discussion

The great amount of data on cancer-related molecular interactions and gene expression patterns has challenged the use of comprehensive information highlighting the multifaceted functions driving tumor progression. In this vein, large-scale informatics studies have provided the chance to handle and analyze the open-source biological datasets. As a better understanding of key regulatory networks involved in cancer biology may strongly boost the identification of new targets and innovative therapeutic approaches, the use of big data regarding the gene expression landscape in cohorts of cancer patients could represent a promising perspective. Considering the aforementioned remarks, the TCGA and the METABRIC datasets were queried to deepen the current knowledge on the action of GPER in BC development. Our data analysis demonstrated that a significant association of GPER with genes belonging to pro-migratory and metastatic signaling pathways occurs in the subset of BC patients lacking ER. Of note, these findings suggest that GPER may be involved in the metastatic dissemination of BC cells in the aforementioned patients. Finally, we ascertained that a worse DFI characterizes the subgroup of both ER and HER2-negative BC patients exhibiting a high expression of GPER, hence highlighting its potential role in the aggressive subtype of breast malignancies.

The action of GPER in mediating the stimulatory effects of estrogens in BC has been extensively reported [6,19,22,23]. In particular, it was established that GPER is involved in a complex transduction network that includes, for instance, the EGFR/MAPK signaling cascade, the adenylyl cyclase, and PI3K, which in turn leads to gene expression changes and biological responses as the proliferation, the survival, and the migration of BC cells [5,6,24]. GPER and ER are considered to be unique estrogen receptors on the basis of their different chromosomal localization and the biochemical, the biological, and the pharmacological properties [19]. Worthy, the observations that BC cells lacking ER may express GPER and that the transcription of the two receptors is differentially regulated in BC phenotypes may indicate an independent beyond a cooperative action of GPER and ER in mediating the estrogen signaling [10,25,26]. In this respect, a physical and functional interaction occurring between these main transduction mediators was shown to regulate transcriptional and biological responses in cancer cells, hence indicating that GPER and ER may synergistically contribute to the malignant progression of estrogen-sensitive tumors [27,28,29,30]. Yet, numerous clinical studies have demonstrated that, in diverse cohorts of BC patients, high GPER levels are likely concomitant with an ER positivity [10,11,31,32]. Nevertheless, immunohistochemical analysis of 361 BC also revealed that the expression of GPER and ER may be not interdependent, as approximately 50% of ER-negative breast tumors retained GPER; therefore, GPER could drive estrogen responses in these peculiar cell contexts [10]. On the basis of these findings, we therefore focused on the gene expression profile and the signaling pathways associated with GPER in ER-negative BC patients. Of note, the expression of pro-metastatic CAMs, ECM-receptor interaction, and FA genes was found as the most correlated with GPER in this cell context, suggesting the potential of GPER to contribute to spreading and metastatic outgrowth of BC cells, as previously reported [7,19,33,34,35,36]. CAMs are cell surface glycoproteins involved in the establishment of normal tissue structure and function, hence contributing to a variety of physiological processes as morphogenesis, embryogenesis, organogenesis, immunological function, wound healing, and inflammation [37]. Cadherins, integrins, selectins, and members of the immunoglobulin superfamily are the four major groups of CAMs mainly involved in transduction signaling, cytoskeletal organization, and gene regulation upon cell-to-cell and cell-to-ECM interactions [38,39]. Hence, alterations in their expression may contribute to peculiar features of neoplastic transformation, including the loss of cellular morphology and tissue architecture [40,41] as well as cell invasion, migration, EMT, trans-endothelial migration, intra- and extra-vasation, tumor angiogenesis, and organ-specific metastasis [42]. In line with our and other previous studies [12,43,44], the present data analysis determined that one of the most GPER-correlated genes belonging to the CAMs pathway is the microvessel density marker *CD34*. As further pro-tumorigenic GPER-associated genes belonging to the CAMs pathway, *CDH5, CLDN*-5, *ESAM*, *CADM3, JAM2,* and *JAM3* were also identified. In particular, *CDH5*, *CLDN*-5, and *ESAM* were indicated as relevant players in BC progression and recurrence [45,46,47,48]. As it concerns *CADM3*, *JAM2,* and *JAM3*, their role in breast malignancies has not yet been elucidated; however, several studies revealed their pro-tumorigenic role in diverse types of malignancy [49,50,51,52]. 

Focal adhesions are protein complexes that connect the cell cytoskeleton to the ECM and then act as scaffolds in outside-in transduction signaling [53,54,55]. In particular, the FAs-mediated intracellular pathways cooperate with receptor tyrosine kinases toward the regulation of cell shape, polarity, adhesion, migration, differentiation, survival, and proliferation [56]. As it concerns cancer development, an altered expression and function of both ECM and FAs has been shown to be crucial for the dissemination of breast tumor cells and therefore for the acquisition of malignant features [9,54,57,58,59,60]. Among the GPER-associated genes belonging to the ECM-receptor interaction and the FA pathway, we found *COL6A1*, *COL6A2,* and *LAMB2* in particular. An increased collagen deposition was shown to exert a fundamental role within the tumor microenvironment toward cancer growth and escape [61,62], and specifically *COL6A1* was involved in both cell proliferation and metastasis of diverse malignancies as BC [63,64,65,66,67]. Likewise, laminins including *LAMB2* have been involved in the maintenance and the regulation of cell polarity, anchorage-independent growth, migration and invasion, EMT activity, metastasis, resistance to anoikis, and a poor outcome in BC [68,69,70,71,72,73,74,75]. Further extending our previous findings [43], the present analysis showed a positive correlation between GPER and *IGF1* expression in ER-negative BC patients. These data fit well with the capability of IGF1 to regulate GPER expression toward BC growth [28,43,76]. Moreover, in the current study, *PDGFRB* was demonstrated as an additional FA gene associated with GPER. Although the role of the PDGFB/PDGFRB axis in BC progression is still a subject of debate, PDGFRB overexpression was correlated with the acquisition of vascular-like functional properties of TNBC, suggesting its involvement in tumor aggressiveness [77,78]. From our correlation and pathway analysis, two members of the ECM-receptor interaction and the FA pathway, named *PGF* and *VWF*, which were indicated as prognostic markers in BC [79,80], appeared to be associated with GPER.

The correlation between GPER expression and clinicopathological determinants of BC progression, including survival, tumor size, number of positive lymph nodes, and vascular invasion, still remains to be understood [11,12,31,32,81,82,83]. In this regard, we ascertained that a high expression of GPER correlates with a short DFI in the aggressive BC lacking ER, hence corroborating our findings on the gene expression profile associated with GPER in this subgroup of patients.

To date, controversial findings on the prognostic role of GPER in BC have been reported. For instance, a recent survival analysis demonstrated an association of high expression of GPER with low overall survival of BC patients [81]. In addition, the expression of GPER was indicated as an independent unfavorable factor for relapse-free survival in BC patients treated with tamoxifen [83]. Accordingly, the involvement of GPER in the resistance to tamoxifen was suggested in previous studies [84,85]. Moreover, immunohistochemical investigations associated the lack of GPER in the plasma membrane with an improved long-term prognosis of tamoxifen-treated patients [86]. Overall, these findings point to the need for a better understanding of the role exerted by GPER in breast tumors.

## 5. Conclusions and Future Perspectives

In the present study, we found a correlation between GPER expression and pro-metastatic genes in ER-negative BC, as assessed querying the TCGA and the METABRIC datasets. In this respect, a deeper understanding of the functional relationships between GPER and these genes would allow the identification of the molecular mechanisms through which GPER may be involved in the aggressive features of breast tumors lacking ER. We also determined that a high expression of GPER correlates with a short DFI in the aforementioned BC subtype; nevertheless, further studies are required to better assess the significance of GPER in breast malignancies characterized by a worse prognosis.

## Supplementary Materials

The following are available online at https://www.mdpi.com/2073-4409/9/3/622/s1, Figure S1: Enrichment plots of KEGG CAMs (A,B) ECM-receptor interaction (C,D) and FA pathway genes (E,F) by GSEA in ER-negative BC patients of TCGA and METABRIC datasets. Enrichment scores (ES) and relative *p*-values are plotted. A positive enrichment score (ES) indicates an enrichment of the selected gene set at the top of the ranked list. The score is calculated by walking down a list of genes ranked by their correlation with the selected phenotype (high or low GPER levels), increasing a running-sum statistic when a gene in that gene set is encountered (each blue vertical line underneath the enrichment plot) and decreasing it when a gene that isn’t in the gene set is encountered, Table S1: Genes correlated with GPER (n. 277) and shared between TCGA and METABRIC datasets.
