# Supplementary material for: The G Protein-Coupled Estrogen Receptor (GPER) Expression Correlates with Pro-Metastatic Pathways in ER-Negative Breast Cancer: A Bioinformatics Analysis
Source: Cells. 2020 Mar 4;9(3):622. doi: 10.3390/cells9030622 (PMC7140398; doi:10.3390/cells9030622)
Supplement: Supplementary file 1 [file cells-09-00622-s001.pdf]

Table 1. Genes correlated with GPER (n. 277) and shared between TCGA and METABRIC datasets.

	TCGA		METABRIC	
**Gene**	**r-value**	***p*-value**	**r-value**	***p*-value**
A4GALT	0.26410409183284	0.00048218759295939	0.178124216445386	0.000158591964358692
ABCA6	0.263056346701776	0.000508754868642476	0.294210449422986	2.45884310323715e-10
ABCA8	0.319646811875607	2.02957245711113e-05	0.298926715743347	1.22769617813487e-10
ABI3BP	0.246934894239449	0.00113001312224866	0.210868279896342	7.24695181443356e-06
ABLIM3	0.24447252327587	0.0012708344991597	0.178701776824928	0.000150851888134839
ABTB1	0.314906073785822	2.72921541366674e-05	0.252437661897648	6.74379123313097e-08
ACACB	0.269206426794594	0.000370190589563145	0.431840682324634	1.21568593554383e-21
ACVRL1	0.272079972197578	0.000318257016497265	0.27693800515491	2.81227684211681e-09
ADAMTSL2	0.244141637247275	0.00129093523780354	0.202381166656779	1.69398807297865e-05
ADCY4	0.372033877119331	5.43399227690584e-07	0.348135246817647	3.99744943833284e-14
ADD1	0.271011119959715	0.000336729271881162	0.255260463189147	4.75345365211831e-08
ADH4	0.275894270240194	0.000259727337378546	0.189258402715319	5.88070692097977e-05
ADRA2A	0.234020673102846	0.00206538338884787	0.215470959851156	4.50596089144891e-06
AFAP1L2	0.308519346291635	4.03572219241505e-05	0.270237339307319	6.92418127730128e-09
AHNAK	0.28928806923352	0.000124258648104014	0.172843043173311	0.000248810040024382
AHNAK2	0.262519032843278	0.000522897923020441	0.295934159272648	1.91040095170126e-10
ANGPTL2	0.245721570058855	0.00119751088020174	0.205125957030751	1.29215371946868e-05
ANKRD29	0.376474571946722	3.87691137533249e-07	0.305970124767284	4.24918777107695e-11
AOX1	0.262310436178499	0.00052848574928274	0.230128199975425	9.25189867671712e-07
APLNR	0.299739949191261	6.81002835148127e-05	0.205081638283272	1.297853065582e-05
AQP7	0.239297373669045	0.00162048581893865	0.305097363331339	4.85375311503133e-11
ARHGAP23	0.327416974034571	1.23549225657156e-05	0.249013354200725	1.02501569221456e-07
ARHGEF17	0.264060109421368	0.000483276543571544	0.180462864492896	0.000129382172133743
ASPA	0.321100571253912	1.85149501392357e-05	0.337913001210092	2.39265574464339e-13
BCHE	0.277961373502021	0.000232353298987716	0.228335533280079	1.12934973846312e-06
BEND6	0.238734174029387	0.00166340934678032	0.242130842678147	2.33436297727138e-07
BGN	0.261371588414254	0.000554324588806297	0.1808532675608	0.000125028329451084
BHLHE41	0.266170490800471	0.000433510205410634	0.17931707478158	0.000142996803489622
C12orf54	0.278592273895634	0.000224548740577043	0.189859176399143	5.56495845444205e-05
C14orf180	0.259746435544915	0.00060182519333257	0.22901963588936	1.0468026284825e-06
C1QTNF1	0.247105827890003	0.00112078931198708	0.208226762946651	9.47451031731385e-06
C1QTNF7	0.317817861799498	2.27660630902962e-05	0.176571958595101	0.00018128032284371
C6	0.296159270541987	8.38992183103835e-05	0.244151616217137	1.83791657012751e-07
C7	0.286409171894513	0.00014605319059461	0.19908160777457	2.3344529127658e-05
CACNA1G	0.255237679420389	0.000753961556819055	0.272601963512439	5.05230882416917e-09
CACNA2D3	0.249450923906171	0.00100102020763697	0.293348307520279	2.78790290906943e-10
CADM3	0.285424391729149	0.000154293151261396	0.278151369260233	2.38264670501991e-09
CALHM2	0.339491913974902	5.5586128149048e-06	0.246274980883833	1.42634110860723e-07
CAV1	0.370599226570178	6.0537813357909e-07	0.183705485945343	9.71541678620513e-05
CCDC85A	0.363581941699127	1.01913066099489e-06	0.201804980867004	1.79223658358192e-05
CD248	0.284497486686933	0.000162443176426094	0.344448263082867	7.67996965125229e-14
CD34	0.299364526452762	6.96153140903943e-05	0.252394201876557	6.77998120002087e-08
CDH5	0.290851154723017	0.00011373777082982	0.212096449997289	6.39043166465618e-06
CDO1	0.302292766260155	5.85898411757477e-05	0.321784879263904	3.53068885040822e-12
CETP	0.237552042101367	0.00175689421586217	0.286145974721282	7.83166091692515e-10
CFD	0.260965011522981	0.000565872140993111	0.223862763697394	1.84431113108548e-06
CIDEA	0.259769109708537	0.000601137400528887	0.348955980405382	3.45261710193374e-14
CIDEC	0.236435216121708	0.00184958713480922	0.279865352780005	1.88259354998361e-09
CLDN5	0.29919288704358	7.03184464266098e-05	0.389960199343345	1.30031791562565e-17
CLEC14A	0.30441620682037	5.16439329423625e-05	0.213128392224365	5.74624843635138e-06
CLEC1A	0.234847620122737	0.00198902572958022	0.182778784428085	0.000105495791679868
CLEC3B	0.347437240551109	3.22600090957855e-06	0.352734087553715	1.74907790217573e-14
CLMP	0.240751384405969	0.00151430024086931	0.327173547245758	1.46197122397692e-12
CNRIP1	0.286535448497846	0.000145026815763766	0.243453953624218	1.99655108179031e-07
CNTNAP1	0.266561319703363	0.00042483127557319	0.308384537185855	2.93416526192429e-11
COL14A1	0.296959947542444	8.00939749785684e-05	0.246871361481752	1.32775150914689e-07
COL16A1	0.26291616608678	0.000512410196054916	0.375132259456508	2.56392708408857e-16
COL17A1	0.313937093051731	2.89776465361125e-05	0.183420509628871	9.96508607169025e-05
COL18A1	0.263387295190976	0.00050022047595038	0.27655234206556	2.96393737032224e-09
COL6A1	0.288471910913691	0.000130106824401569	0.248973142183286	1.03003010548523e-07

COL6A2	0.299869496108426	6.75847078188505e-05	0.284832360046129	9.42557472122505e-10
COL7A1	0.314330966466173	2.82810262869742e-05	0.2631008202356	1.76003840557806e-08
CORO2B	0.3307286934492	9.95764133637597e-06	0.3291451467805	1.05421475873619e-12
COX4I2	0.259204195518468	0.000618491183921994	0.257636214442735	3.52985532060192e-08
CPS1	0.234440296662876	0.0020263098044302	0.234935653545614	5.37669029417577e-07
CSRNP1	0.249498700108605	0.000998707079979917	0.240649050520646	2.77800645967445e-07
CST3	0.315249252634844	2.67176921291732e-05	0.176915627379626	0.000176009446986487
CTSG	0.346012073439402	3.5606395639354e-06	0.227905360591495	1.18441536878289e-06
CYGB	0.28176729126101	0.000188841897982313	0.26781918593248	9.52764414219325e-09
CYP11A1	0.290352170502017	0.000117002286396882	0.205663020052717	1.22495253705324e-05
CYTL1	0.295397805728384	8.76746143903088e-05	0.206996357606913	1.07218971020214e-05
DACT3	0.235822670727403	0.00190229671531381	0.220482553114765	2.65416264736593e-06
DGKA	0.254446032181448	0.000784072255339209	0.177118473417858	0.000172966146500497
DLC1	0.265598884043901	0.000446499317231834	0.356500014923089	8.79950851959844e-15
DLG4	0.254797380780568	0.000770574553880767	0.343701108256284	8.75731204918098e-14
DLK2	0.273309113957808	0.000298178610458716	0.246477987043647	1.39201466181074e-07
DLL1	0.256368287513496	0.000712800690329784	0.233787386265841	6.1274374655315e-07
DNAH1	0.245445637098954	0.00121336751481988	0.225166701632213	1.60024600931563e-06
DOCK6	0.25159256793929	0.000902020109016834	0.184865576806847	8.75853965671839e-05
DOK4	0.26688475902407	0.000417770726355042	0.199547378010514	2.23183860680472e-05
DSEL	0.259389669559441	0.000612743254554438	0.336391852262937	3.1052241322798e-13
EBF1	0.298570829470486	7.29229353131299e-05	0.354798115171855	1.20165977675218e-14
EBF2	0.336538655265665	6.7788671776341e-06	0.31701076357911	7.59955441588546e-12
EBF3	0.31423914470629	2.84420011066983e-05	0.312080401696101	1.65365404123425e-11
EFNB1	0.333765900603203	8.15226670362772e-06	0.334618693983283	4.20026381020701e-13
EFNB3	0.248528556450015	0.00104665204564711	0.201233608839034	1.89499082527091e-05
EGR3	0.286881179571726	0.000142251056617902	0.18114657910735	0.000121848225243819
EHD2	0.308870749945604	3.95070973004215e-05	0.370057711115082	6.87152202462454e-16
EMID1	0.241470506462421	0.00146416633627349	0.225750025149226	1.50137368347633e-06
EMILIN1	0.295302500387991	8.8158189529875e-05	0.243762404692355	1.92485317462802e-07
EML3	0.284614464969525	0.000161392926159812	0.292597682248245	3.10899599214925e-10
ESAM	0.266564157473589	0.000424768850904346	0.220391774550906	2.68002726782199e-06
ESPNL	0.234551773476455	0.00201604318715485	0.268128642108871	9.1479320800942e-09
EVL	0.290716377553766	0.000114611031182782	0.178460131832028	0.000154046104664464
F10	0.302804411316778	5.68402677307817e-05	0.206431598779247	1.13453998180916e-05
FABP4	0.293576642543872	9.73590019848194e-05	0.293748554663816	2.63012965367656e-10
FCER1A	0.266205123260382	0.000432734567379193	0.189568778275264	5.71552980398958e-05
FEZ1	0.306179709793199	4.64711995645708e-05	0.309212234067874	2.58233819444093e-11
FHL1	0.245746836653237	0.0011960684451643	0.347375835302527	4.57611951391863e-14
FHOD3	0.235123294748055	0.00196414729787173	0.218447810227419	3.29536356375285e-06
FLRT2	0.332564587210564	8.82575184984601e-06	0.336235721193133	3.18917163843001e-13
FOXS1	0.284343668423434	0.000163833883330975	0.20056039600861	2.02323682240074e-05
FSCN1	0.239100626426153	0.00163536446863527	0.241247884818291	2.58972956458343e-07
FXYD1	0.271417347473532	0.000329594627410803	0.295824880282209	1.94131075343837e-10
GAS6	0.328334161620301	1.16413426136917e-05	0.231879810106883	7.60214281988999e-07
GDF10	0.26371305810222	0.000491949177125538	0.188215526083606	6.46932106302802e-05
GFRA2	0.258082387904654	0.000654329621330947	0.327240324809424	1.4459250261018e-12
GJA4	0.270527940618881	0.000345401827198035	0.18910434731266	5.96435614902169e-05
GJC2	0.277303715288856	0.000240756887948801	0.231338604158829	8.07908239627607e-07
GLI2	0.267996228992346	0.000394327281219917	0.200276907952545	2.07966897293842e-05
GNG11	0.302570119731124	5.7635252531309e-05	0.192661273359584	4.29232358666335e-05
GPIHBP1	0.296655754573656	8.15202064913102e-05	0.293099963785701	2.89038832683667e-10
GPR146	0.350487472051538	2.60745894208097e-06	0.217338146696351	3.70491620214498e-06
GPR162	0.255583663767623	0.000741139120251567	0.407029663904331	3.46781963193149e-19
GRASP	0.326013022382309	1.35278260198016e-05	0.286220862436801	7.74916119058023e-10
HEPACAM	0.302839510926813	5.67220600924342e-05	0.274120091167109	4.12017594431454e-09
HEPH	0.25401223910753	0.000801037173025339	0.227026743892697	1.30499187996507e-06
HEYL	0.248679022307239	0.00103908024172257	0.173460840387862	0.00023619854274676
HHEX	0.265539769190367	0.000447862977251875	0.186481583218613	7.57264698887116e-05
HIC1	0.316828403117579	2.42180837967373e-05	0.188438193935217	6.339160773466e-05
HIGD1B	0.239956760876682	0.00157151673379751	0.187034376938649	7.20297025035026e-05
HRC	0.246392303838968	0.00115975474114503	0.216303185019528	4.13038273248633e-06
HSPB6	0.24937163283319	0.00100487000680683	0.35837207529192	6.23251789440982e-15

HSPB7	0.243149867345013	0.00135293838322521	0.212596618191748	6.07005038776689e-06
HSPG2	0.249084410331289	0.00101892944752755	0.188788632052899	6.13930484309835e-05
HYAL1	0.267544571701209	0.000403702825495956	0.253935213029136	5.60463764838982e-08
ID1	0.243387202603524	0.0013378578293016	0.284648309485851	9.67268189739571e-10
IFFO1	0.238579218684591	0.00167539957351653	0.233967550426378	6.00332477078319e-07
IGDCC4	0.344231926108718	4.02507138833729e-06	0.29368246465324	2.6555688475808e-10
IGF1	0.303798272861183	5.35810138518382e-05	0.174263790827185	0.000220699735072662
IGFBP4	0.344981231873317	3.82297141252248e-06	0.203964068008749	1.44972842846951e-05
IGFBP6	0.330432831694055	1.01524363462649e-05	0.275779613432257	3.29205738730521e-09
IQSEC1	0.246148948052649	0.00117332555105289	0.203296029402838	1.54842547343389e-05
ITGA5	0.266440320204483	0.000427500908881926	0.207719603338901	9.97094259316735e-06
ITGA7	0.267077125849786	0.000413623104678541	0.201946042611166	1.76769508648112e-05
ITIH5	0.314786749714326	2.74946085196717e-05	0.174886501402491	0.000209340743527191
JAG1	0.271750474566732	0.000323848760232089	0.218240399186382	3.36846994964171e-06
JAM2	0.291061794744683	0.000112385420122257	0.211754336632184	6.61878950152302e-06
JAM3	0.272065033892452	0.000318508576426532	0.26034991293484	2.50340291275846e-08
KANK3	0.356559622596877	1.69545024876081e-06	0.208699698752481	9.03285413672773e-06
KCNJ8	0.265424811517844	0.00045052585411248	0.29087361796732	3.98873387273141e-10
KIF17	0.267082846620035	0.000413500344756373	0.193221032396354	4.07353196229804e-05
KLHL29	0.269255092121649	0.000369249238836372	0.229289802396909	1.01582421536287e-06
LAMA2	0.349113955940852	2.8705556977062e-06	0.212132378732004	6.36689028146817e-06
LAMB2	0.290611041525059	0.000115297892676465	0.229406738126284	1.00269032008075e-06
LDB2	0.266300114665333	0.00043061370004385	0.269746331877187	7.3896826487003e-09
LMOD1	0.244486886518548	0.00126996847782087	0.176700734208327	0.000179288188938686
LRP1	0.279878809094563	0.000209383583852305	0.298349372599939	1.33755637828644e-10
LRRC17	0.256374410762304	0.000712583513539653	0.217911043885452	3.48775244259944e-06
LRRN4CL	0.277958087866519	0.000232394596368418	0.286782313904218	7.1569865552238e-10
LTBP4	0.318718410045641	2.15162516018324e-05	0.223822115684304	1.85246497695454e-06
LYL1	0.267737071354419	0.000399681945157528	0.2372132416512	4.14057883774442e-07
MAN2C1	0.239469643789414	0.0016075597074537	0.222543232317949	2.1273711806276e-06
MAP1A	0.248112703003891	0.00106784272117345	0.354431142728573	1.28485480474658e-14
MAP4	0.242411548166069	0.00140085122382212	0.203042120229238	1.58758670869798e-05
MAPKBP1	0.2771902537849	0.00024223497735243	0.214554095312993	4.9574344883072e-06
MEOX1	0.292682312691856	0.000102473044131094	0.24617257363353	1.44396541126866e-07
MEOX2	0.358077594608359	1.52035952512459e-06	0.283591711705027	1.1217855775125e-09
MFAP4	0.280233460164714	0.000205373933892445	0.340520368845929	1.52535735501204e-13
MICALL2	0.254197307317155	0.00079375859374796	0.235907674728316	4.81100746577959e-07
MMP28	0.39015290648784	1.32715609162223e-07	0.255182599173492	4.79980447013875e-08
MRAP	0.235589342744421	0.00192273160330526	0.311833748136063	1.71859396649322e-11
MRGPRF	0.292001189478277	0.000106535306411138	0.311744642812389	1.74266054957807e-11
MYH11	0.282232970356828	0.000184073467665195	0.208059320150014	9.63574965918065e-06
MYL3	0.248168599809539	0.00106497168207978	0.220064454050571	2.77530370779864e-06
MYOZ3	0.268416063422649	0.000385793533012269	0.282244021545354	1.35398587673651e-09
NAALADL1	0.315342731833426	2.656319938877e-05	0.212906713526327	5.8791756527051e-06
NAP1L3	0.247844244859129	0.00108173072843158	0.198229673001838	2.53382236782394e-05
NAV3	0.351454964802418	2.43608307309317e-06	0.177739173358002	0.000163957327277245
NCKAP5L	0.255201919453819	0.000755298423450505	0.179539728239294	0.000140250176387838
NDN	0.305986868440466	4.70121711164242e-05	0.195647829254686	3.24126777482626e-05
NISCH	0.30344501726515	5.47188286632437e-05	0.351713023522633	2.10386693822738e-14
NKD2	0.261748555022908	0.000543812757611304	0.202288754616208	1.70939361559001e-05
NLGN2	0.264002578203401	0.000484704377447227	0.286564201942231	7.38161370664107e-10
NNAT	0.238327966970614	0.00169500864714385	0.20146324724923	1.8530385046346e-05
NOVA1	0.273345000702677	0.000297610453892679	0.20749703882885	1.01965296623496e-05
NPR2	0.273782888363289	0.0002907583067591	0.253882965229565	5.64104678371016e-08
NRIP2	0.326842980349501	1.28223308589991e-05	0.194284411655589	3.68662417450992e-05
NRXN2	0.295094836888016	8.92205270534643e-05	0.251246198375944	7.80677192169454e-08
NTRK2	0.278568784001085	0.000224834897042905	0.256785852108544	3.92798246524409e-08
OLFML1	0.237238583170789	0.00178247342623252	0.250218973931977	8.85150821057405e-08
OPRL1	0.253897025911684	0.000805599362651397	0.178680573382427	0.000151129661887738
PALM	0.279234249072447	0.000216858061459017	0.298688977253138	1.27182653218839e-10
PAPLN	0.236078215062303	0.00188014308189628	0.283340713954176	1.16187619651535e-09
PCDH18	0.260313860024644	0.000584829462669229	0.213195247820907	5.70672520569974e-06
PCDH19	0.260645223528246	0.000575110272626769	0.211390188937964	6.87039456588611e-06

PCSK5	0.293187081918032	9.95565456477033e-05	0.326412359933253	1.65764329954294e-12
PDE1A	0.245379315601057	0.0012172072558755	0.344872102504523	7.12772117181832e-14
PDE2A	0.35551676213442	1.82669388244666e-06	0.358758344207461	5.80273380056552e-15
PDE7B	0.24399439486102	0.0012999731081399	0.298518248769378	1.30446745930946e-10
PDGFRB	0.292197765176049	0.000105347717920993	0.2346507770576	5.55424291423692e-07
PGF	0.301650652183547	6.0857100867987e-05	0.232186765900762	7.34377498008997e-07
PHLDB1	0.281455434688253	0.000192099287483369	0.297662343755035	1.48080171829108e-10
PI16	0.300564208436284	6.48817477163944e-05	0.200145618460906	2.10630698293033e-05
PKD1	0.234663935452469	0.00200576123596962	0.276264593633336	3.08223341099846e-09
PLAC9	0.318838545197017	2.13545033561968e-05	0.321546376577793	3.66971300299694e-12
PLCH2	0.283056600358282	0.000175913322471078	0.274886897623104	3.71509161236073e-09
PLVAP	0.284169340898034	0.000165423436803827	0.200235564439983	2.08802269870705e-05
PLXDC1	0.282255560087884	0.000183845032477666	0.233825659047867	6.10086739891655e-07
PNMA2	0.24194757867086	0.00143174846216528	0.237821887851153	3.85964836515646e-07
PODN	0.257120283696917	0.000686580352629117	0.185775123453524	8.07114376646548e-05
POLR3GL	0.237261743388313	0.00178057193721341	0.176087756872164	0.000188957954657339
PRDM8	0.268360367923596	0.000386915700176508	0.245799342616615	1.50999234168797e-07
PRG4	0.278710996680438	0.000223107623202396	0.201891550144139	1.77713745458005e-05
PROCR	0.325160458253045	1.42906487950917e-05	0.196063943641776	3.1158263777255e-05
PROS1	0.254626416748429	0.000777115517861759	0.246402445521146	1.40469395265132e-07
PTGS2	0.237609357025234	0.00175225347321085	0.232544224755362	7.05351994109656e-07
PTH1R	0.337169029209425	6.4988094700171e-06	0.481610777934092	3.21017273379166e-27
PTPRB	0.241609148056569	0.00145467697404683	0.190724892209888	5.13799974643947e-05
RAMP2	0.294229274372382	9.37788707304417e-05	0.22856979531569	1.1004036089715e-06
RAMP3	0.357943343669845	1.53512181038694e-06	0.200253348112709	2.08442549721344e-05
RAPGEF3	0.25076648578027	0.000939089400354932	0.313066982390146	1.4170236130374e-11
RECK	0.240314485180084	0.00154551914873445	0.224551641316265	1.71124237567915e-06
RFX2	0.23802382659259	0.00171902525977312	0.194897838543501	3.47952367452102e-05
RGS12	0.296560027711238	8.19739275683262e-05	0.262212354200752	1.97301911842648e-08
RHOJ	0.276346007826157	0.000253500989732773	0.226095840007541	1.44555129222466e-06
RIN1	0.264526597622202	0.000471841803252308	0.220105570348152	2.76316007296798e-06
RIPK3	0.245643491692336	0.00120197833778871	0.191860730567836	4.62460998458145e-05
ROBO3	0.335106886479027	7.45806165015416e-06	0.215679565464885	4.40886823123932e-06
ROBO4	0.293144915021093	9.97971760286683e-05	0.228669303213919	1.08832462898771e-06
RUNX1T1	0.234404090612968	0.00202965446733588	0.242063955434473	2.35282658758106e-07
S1PR1	0.265837897737725	0.000441024819199149	0.193576225184055	3.94021422919037e-05
SCARA5	0.253364888931401	0.000826982507668945	0.248461673557338	1.09591108143455e-07
SCN1B	0.323653031735285	1.57398440245766e-05	0.252404100198409	6.77172223163504e-08
SCN4A	0.328946751232046	1.11866529252634e-05	0.280521873894654	1.7194294879751e-09
SCN4B	0.364520287800969	9.51242868122345e-07	0.306605699984262	3.85579316316461e-11
SEMA3G	0.299631754470171	6.85337050498837e-05	0.300598255242702	9.5688492266919e-11
SEMA5A	0.29015267254287	0.000118331805496836	0.21272794376502	5.98850424712127e-06
SEMA6B	0.243002935317791	0.00136235233592773	0.300117105064302	1.02821798976771e-10
SEPT4	0.247761131824718	0.00108606381899876	0.184944014453981	8.69715455703189e-05
SGCA	0.301247141807869	6.23237181966275e-05	0.205638193119384	1.22798436616084e-05
SH2D3C	0.260892464775357	0.000567955800643792	0.224095203657424	1.79834064728745e-06
SH3PXD2A	0.241769553182988	0.00144376811615585	0.291517667888331	3.63492089643067e-10
SH3TC1	0.249987096010912	0.000975340678541621	0.187460222001155	6.92986173924198e-05
SHANK3	0.258279192672795	0.000647907005871092	0.221010557895354	2.50839220733844e-06
SHE	0.250972629322964	0.000929710266029302	0.217284984916031	3.72571021892728e-06
SLC12A4	0.235027081631944	0.00197279776683972	0.17533382065897	0.000201521391784239
SLC17A7	0.357282921153119	1.60975242881534e-06	0.178234628368806	0.000157084086372283
SLC27A3	0.262269533521257	0.000529587884010346	0.340154762935129	1.62516265403502e-13
SLC38A11	0.266104884873825	0.00043498306116196	0.231383643500619	8.03832037106225e-07
SLIT3	0.250665233578703	0.000943727923646246	0.2948958718747	2.22450966756807e-10
SNRK	0.235269619608965	0.00195105744680524	0.230681993015779	8.69634315351211e-07
SOX15	0.267480808084836	0.000405042937936023	0.18801965781353	6.58589585550867e-05
SOX17	0.261818217407552	0.0005418904661963	0.189131893374689	5.94931717701447e-05
SOX18	0.245748657504522	0.00119596455675186	0.179637619008328	0.000139058347622561
SRL	0.332743972102746	8.72194200684288e-06	0.298046159119682	1.39903012509964e-10
SSPN	0.264718751685299	0.000467204803853885	0.208406552043099	9.30424320186035e-06
ST3GAL2	0.336310286176139	6.88311958668045e-06	0.17745601430864	0.000168011093778821
ST6GALNAC6	0.261403205237187	0.000553435789838905	0.265165603713348	1.34748151928716e-08

STARD8	0.251809770610443	0.000892499150412194	0.352587748029696	1.79606733730154e-14
STK32B	0.295009958385528	8.96581780243688e-05	0.228741434467804	1.07964842994652e-06
SVEP1	0.287025883583489	0.000141104063321548	0.241699657177201	2.45588240971618e-07
SYDE1	0.270461221881856	0.000346615497680897	0.219229202055748	3.03333077229825e-06
TBX15	0.259372041403559	0.000613287433368911	0.243480299768602	1.99032793790783e-07
TBX2	0.260435307067636	0.000581249853229684	0.281059275224706	1.59616943945767e-09
TEK	0.28083528240208	0.000198732947920719	0.188570954999967	6.26273521037446e-05
TGFB1I1	0.297842822330882	7.60858028791542e-05	0.211764525245748	6.61187769248551e-06
THSD1	0.338292262838002	6.02677178033785e-06	0.197141550906683	2.81199519616241e-05
TIE1	0.311470328561716	3.3721782560168e-05	0.18836815252528	6.37983468007702e-05
TIMP4	0.244444959005392	0.00127249798053505	0.312850997982137	1.46582097274132e-11
TMEM115	0.245095951195368	0.00123373849444053	0.181611325392147	0.000116964586416853
TNK2	0.270354227518275	0.000348570072468172	0.196229943260156	3.06707882449158e-05
TNNT3	0.246693217729844	0.00114317302171912	0.195443049294845	3.3047419364992e-05
TP63	0.238456583306421	0.00168494472978057	0.226908877356841	1.32203581597163e-06
TPST1	0.273112478365806	0.000301309644445681	0.205158520820529	1.28798122448358e-05
TRPC1	0.323696351595036	1.56963269537096e-05	0.289887821340735	4.59604258967195e-10
TSHZ3	0.254549405334148	0.000780078575117079	0.18934785672671	5.83264446064024e-05
TSPAN4	0.237224490976973	0.00178363132179593	0.19593698711746	3.15360270817859e-05
TSPAN7	0.271536823871317	0.000327523078851291	0.25852994307787	3.15353875882163e-08
TWIST2	0.276706412285157	0.000248633293554055	0.288509154616223	5.5984062770052e-10
VILL	0.297925256241182	7.57213152578118e-05	0.211519562585873	6.77997826522036e-06
VWF	0.273913189637805	0.000288747782622487	0.321550122031421	3.66748897462522e-12
XPNPEP2	0.274644701297494	0.000277698293866361	0.279740714053208	1.91522414556882e-09
YPEL4	0.252721217058368	0.000853544450722531	0.305347468149543	4.67241914621413e-11
ZBTB4	0.270469469893338	0.000346465245479912	0.245631421455944	1.54063899620742e-07
ZBTB47	0.261183410276169	0.000559641891286031	0.248465497533092	1.09540376994007e-07
ZCCHC24	0.248215393448211	0.00106257365490192	0.309322050475922	2.53886889125071e-11

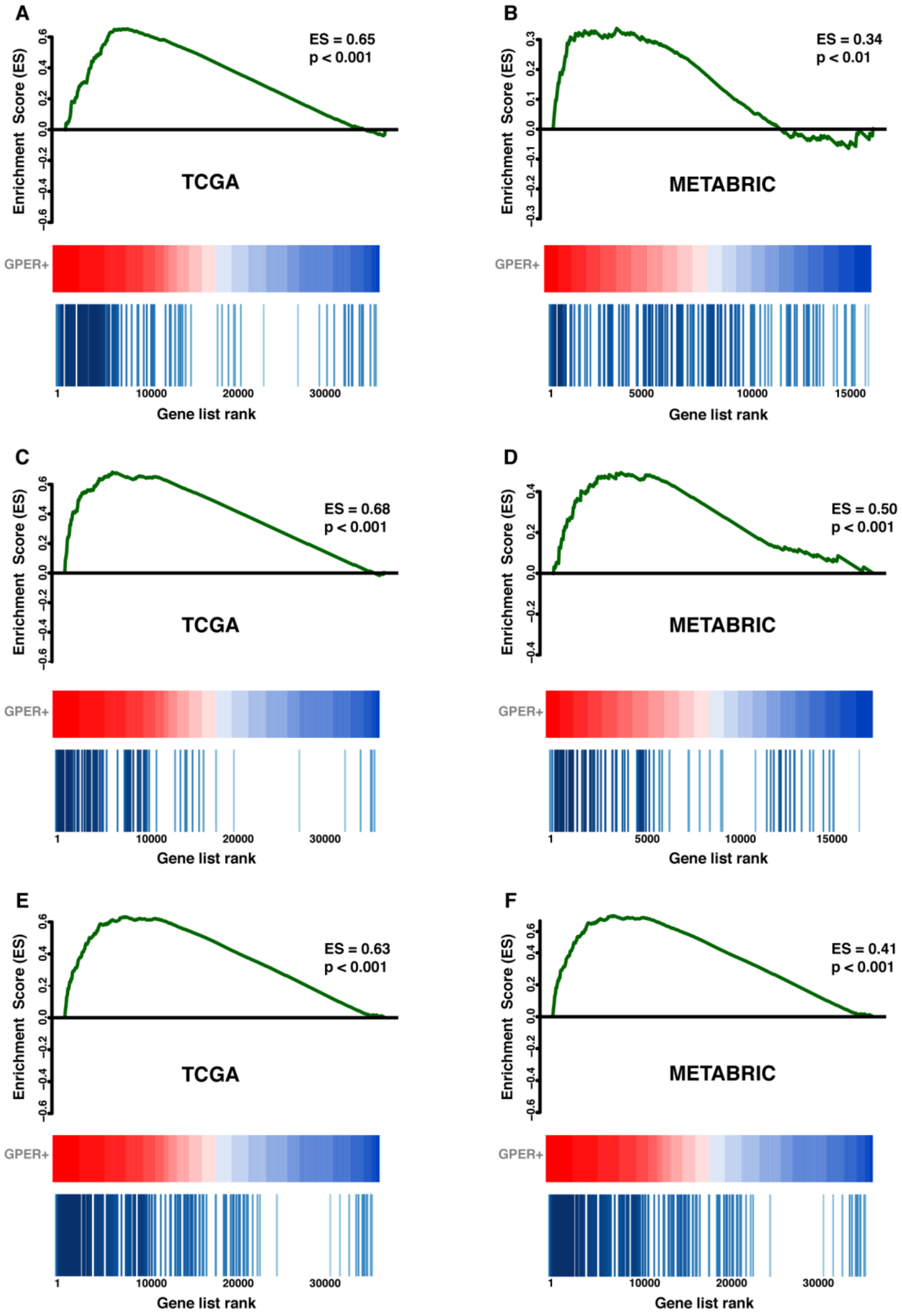

Figure S1. Enrichment plots of KEGG CAMs (A, B) ECM-receptor interaction (C, D) and FA pathway genes (E, F) by GSEA in ER-negative BC patients of TCGA and METABRIC datasets. Enrichment scores (ES) and relative p-values are plotted. A positive enrichment score (ES) indicates an enrichment of the selected gene set at the top of the ranked list. The score is calculated by walking down a list of genes ranked by their correlation with the selected phenotype (high or low GPER levels), increasing a running-sum statistic when a gene in that gene set is encountered (each blue vertical line underneath the enrichment plot) and decreasing it when a gene that isn't in the gene set is encountered.
